# Ratio-based multi-level resistive memory cells

**DOI:** 10.1038/s41598-020-80121-7

**Published:** 2021-01-14

**Authors:** Miguel Angel Lastras-Montaño, Osvaldo Del Pozo-Zamudio, Lev Glebsky, Meiran Zhao, Huaqiang Wu, Kwang-Ting Cheng

**Affiliations:** 1 Instituto de Investigación en Comunicación Óptica, Facultad de Ciencias, Universidad Autónoma de San Luis Potosí, San Luis Potosí, México; 2 Institute of Microelectronics, Tsinghua University, Beijing, China; 3 School of Engineering, Hong Kong University of Science and Technology, Clear Water Bay, Kowloon Hong Kong

**Keywords:** Information technology, Electrical and electronic engineering, Electronic devices

## Abstract

Ratio-based encoding has recently been proposed for single-level resistive memory cells, in which the resistance ratio of a pair of resistance-switching devices, rather than the resistance of a single device (i.e. resistance-based encoding), is used for encoding single-bit information, which significantly reduces the bit error probability. Generalizing this concept for multi-level cells, we propose a ratio-based information encoding mechanism and demonstrate its advantages over the resistance-based encoding for designing multi-level memory systems. We derive a closed-form expression for the bit error probability of ratio-based and resistance-based encodings as a function of the number of levels of the memory cell, the variance of the distribution of the resistive states, and the ON/OFF ratio of the resistive device, from which we prove that for a multi-level memory system using resistance-based encoding with bit error probability *x*, its corresponding bit error probability using ratio-based encoding will be reduced to $x^2$ at the best case and $x^{\sqrt{2}}$ at the worst case. We experimentally validated these findings on multiple resistance-switching devices and show that, compared to the resistance-based encoding on the same resistive devices, our approach achieves up to 3 orders of magnitude lower bit error probability, or alternatively it could reduce the cell’s programming time and programming energy by up 5–10$\times$, while achieving the same bit error probability.

## Introduction

Resistive random-access memory (ReRAM) is a promising non-volatile memory technology for the next generation of high-performance and large capacity memories^1^. A ReRAM device encodes information by modulating the electrical resistance of a thin oxide layer that is sandwiched between two electrodes. The physical resistance-switching mechanism in ReRAM devices have been described by the partial formation and destruction of a conductive filament (CF) in the oxide layer^2–4^, and multiple resistance levels have been experimentally observed^5–9^, which opens up the possibility of multi-level cells (MLC) as well as analog-based computing^9–14^.

A major hurdle of this technology has been the large device-to-device and cycle-to-cycle variations^7,9,15^, that are due to the intrinsic stochasticity in the formation and destruction of the CF^16^. Such variations manifest in a large resistance distribution of the memory states, which is particularly problematic for MLC memories that require tight state distributions to reliably pack as many levels as possible into a memory cell. Extensive efforts have been made to address ReRAM device variations through multiple approaches, spanning from device and circuit improvements^17–21^, the use of a current compliance during the formation and destruction of the CF^22–24^, to higher level write-verify programming schemes based on iterative algorithms to achieve high-precision state tuning^6,7,9,25^.

**Figure 1:**
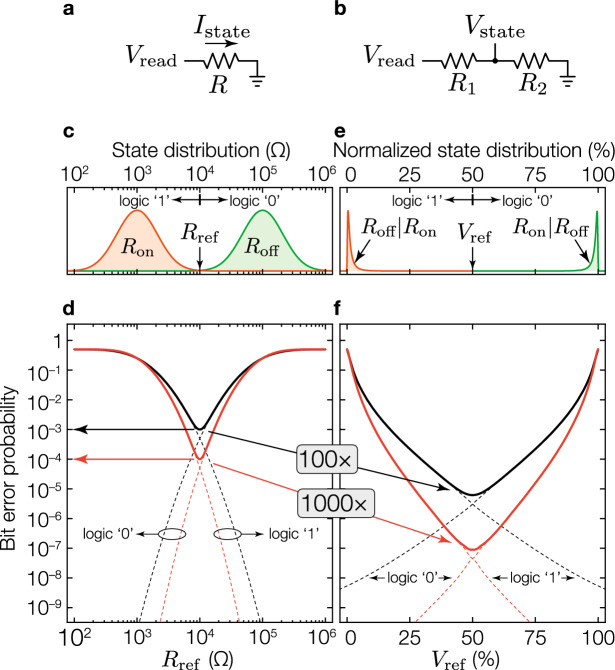
Memory encoding comparison in a two-level cell. (**a**) Configuration used to encode and read a cell using ReBE. (**b**) Voltage divider configuration used to encode and read a cell using RatioBE. (**c**) State distribution of the lowest ($R_\text {on}$) and highest ($R_\text {off}$) resistance states. **d**, BEP using the traditional resistance-based approach as a function of the reference in ohms. (**e**) State distribution under RatioBE. (**f**) BEP for the RatioBE as a function of the normalized reference. For both (**d**) and (**f**) the dashed lines represent the expected individual bit error probabilities for logic ‘1’ and logic ‘0’ (assuming equal probability of storing a logic ‘1’ and a logic ‘0’), whereas the solid lines the total BEP. The black and red set of lines assumes a device variability that produces a minimum BEP of $10^{-3}$ and $10^{-4}$, respectively.

To address the variability of MLC ReRAM devices, our proposal generalizes the idea in^26^ which uses the *ratio of two devices’ resistances*, rather than one device’s absolute resistance (Fig. 1a), to encode information. Specifically, we use two serially-connected resistance-switching devices configured as a voltage divider (Fig. 1b). The state of the memory cell is determined by applying a non-destructive read voltage $V_\text {read}$ across the voltage divider and comparing the voltage at the mid-point ($V_\text {state}$) to a reference voltage $V_\text {ref}$. The proposed solution is orthogonal to the mentioned device and circuit solutions, and in fact it greatly benefits from any improvements due to these sources.

**Figure 2:**
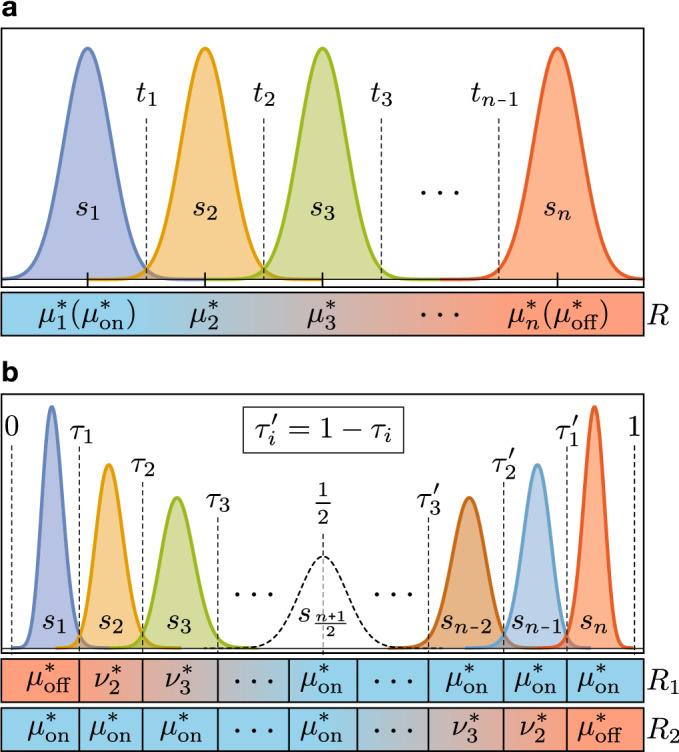
State distribution and threshold location for a multilevel memory. (**a**) Resistance-based states, using a single log-normally distributed resistive device to encode information. (**b**) Ratio-based states, using a pair of devices to encode information. The shape of the distributions is produced using equations (SM. 1.2) and (SM.1.4) (located in Supplementary Note 1) with optimal parameters located in Supplementary Note 3.

**Figure 3:**
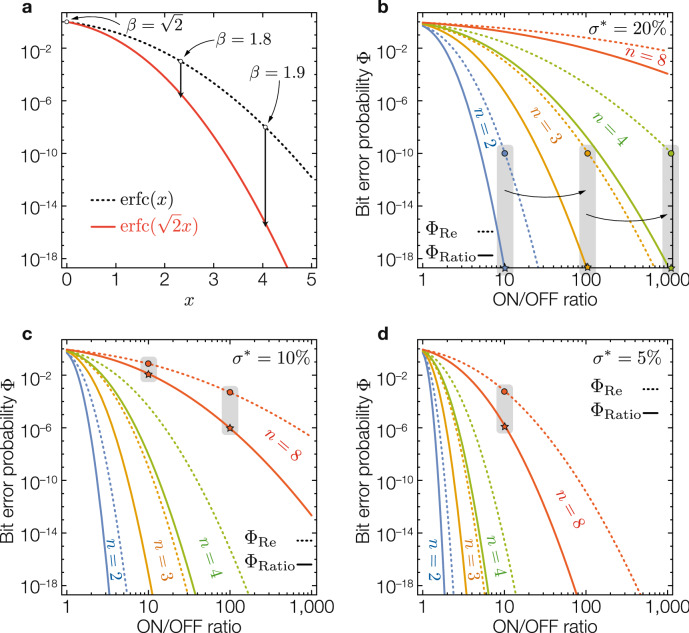
Bit error probability under different parameters. (**a**) Error function complement with a log-normal scale. (**b–d**) BEP for a geometric standard deviation of 20%, 10% and 5%, respectively, for 2, 3, 4 and 8 levels. Note that since $\sigma ^*$ is a multiplicative factor, a value of $X\%$ can be implicitly understood as $1+X$ (e.g for $\sigma ^* = 10\%$ we use $\sigma ^* = 1.10$).

To illustrate the potential of our ratio-based encoding (RatioBE) against the traditional resistance-based encoding (ReBE) in terms of the bit error probability (BEP), we first use a two-level resistance-switching device. Assume that the lowest ($R_\text {on}$) and highest ($R_\text {off}$) resistance states of the device are log-normally distributed and centered in 1 k$\Omega$ (logic ‘1’) and 100 k$\Omega$ (logic ‘0’), respectively (Fig. 1c). Further defining a resistance reference $R_\text {ref}$, we estimate the BEP by deriving the probabilities $\Pr (R_\text {on}>R_\text {ref})$ and $\Pr (R_\text {off}<R_\text {ref})$. Figure 1d shows with dashed lines such probabilities and with a solid line their sum, as a function of $R_\text {ref}$. The variances of the $R_\text {on}$ and $R_\text {off}$ state distributions were assumed identical and so chosen to produce a minimum BEP of $10^{-3}$ and $10^{-4}$ for the black and red lines, respectively. If we connect two such devices ($R_1$ and $R_2$) as a voltage divider (Fig. 1b) where one device is in $R_\text {on}$ and the other device is in $R_\text {off}$ (i.e. either in a $R_\text {on} | R_\text {off}$ or $R_\text {off} | R_\text {on}$ configuration), the distributions of the normalized output of the voltage divider ($V_\text {state}/V_\text {read}$) becomes much tighter (Fig. 1e), and thus are significantly more robust for information encoding. Note that using the RatioBE not only reduces the BEP (e.g. $10^{-5}$ vs $10^{-3}$ for ReBE), but also the reduction would be more significant as the device quality improves (e.g. as the variances of $R_\text {on}$ and $R_\text {off}$ states reduce). For example, if a device improvement achieves a 10$\times$ BEP reduction from $10^{-3}$ to $10^{-4}$ under ReBE, the same level of device improvement will lead to 100$\times$ improvement from $10^{-5}$ to $10^{-7}$ under RatioBE (compare solid lines in Fig. 1d and f). The lower BEP achieved by RatioBE should be intuitive as, under RatioBE, an error occurs only when the resistance of the $R_\text {on}$ device becomes larger than that of the $R_\text {off}$ device in the voltage divider. The probability of such an event is much lower than the probability of a $R_\text {on}$ (or $R_\text {off}$) device’s resistance to be higher (or lower) than $R_\text {ref}$, for which an error occurs under ReBE.

## Multi-level memory cells

A MLC is capable of storing multiple bits of information in a memory cell, effectively increasing the memory’s storage capacity without proportionately increasing the memory’s die area^27^. Regardless of the physical mechanism used to encode the information, an *n*-level cell can encode $\log _2(n)$ bits per cell, and needs $n-1$ references to distinguish the *n* levels.

For a resistance-based MLC, the information is encoded based on the resistance of a single device (i.e. ReBE), as such, the resistance values of the levels and their references are relatively straightforward to determine. For example, if the device has the same variance across all targeted resistance values, the optimal values for the *n* levels and their optimal references would be uniformly distributed between the minimum and maximum resistance values (see for instance Fig. 2a). This is not true, however, for a ratio-based MLC which consists of two devices per cell, namely $R_1$ and $R_2$. When determining the target mean resistance values of these two devices for an optimized MLC, the objective is to maximize the separation of the resulting *n* ratio-based states while minimizing the variances of these *n* states. Since the probability distribution of a state *s* under RatioBE depends on the distributions of $R_1$ and $R_2$ according to $s=R_2/(R_1+R_2)$, the *n* states would have different variances, even when $R_1$ and $R_2$ have the same variance for any target resistance value they are programed to (compare Fig. 2a and b), which makes the search for optimal references ($\tau _i$ and $\tau _i'$ in Fig. 2b) a non-trivial problem.

We focus our analysis on three figures of merit: (1) The probability $\Phi$ of erroneously encoding a bit (BEP), (2) the memory capacity, which is proportional to the number of levels *n* per memory cell, and (3) the *programming effort* or *PE*, which is a measure of the amount of energy and/or time employed to program the devices in the memory cells. Assuming an iterative write-verify programming scheme, the *PE* is proportional to the number of iterations used to program the device, but inversely proportional to the variability of the device’s resulting state, i.e., a higher *PE* increases the programming time and/or energy, but results in sharper distributions of the device’s states^28^.

Given a set of physical device parameters, such as the device’s lowest and highest resistance values ($R_\text {on}$ and $R_\text {off}$), their ratio (i.e. ON/OFF ratio) $\alpha$, and their *intrinsic* variability (represented as the standard deviation $\sigma$ of the states of the device), we need to address a series of design issues and tune specific parameters for designing an optimized ratio-based MLC system. In particular, we need to decide (1) whether to dynamically program $R_1$ and $R_2$, or only one of them, (2) the mean values $\mu _1$ and $\mu _2$ for $R_1$ and $R_2$, respectively, for each ratio-based state, (3) the target level of *effective* variability $\sigma _e$ needed for the devices, which is a function of the *PE*, and (4) the references for differentiating the ratio-based states.

Our approach can systematically and optimally determine these key physical and design parameters, obtaining an optimal configuration with respect to a given figure of merit. For instance, for a target $\Phi$, maximize *n*; for a given *n*, minimize $\Phi$; or for a given *n* and target $\Phi$, minimize the *PE*. We employ a rigorous mathematical approach that results in a compact model for $\Phi$ that enables (1) a quantitative comparison of how much better the proposed RatioBE is against the traditional ReBE, and (2) the effect assessment that each parameter has on the BEP, and the relationship among them. Our model reveals that under log-normally distributed resistance states, for a given $\Phi$ and *PE*, a ratio-based MLC can achieve 20–40% higher memory capacity compared to a resistance-based MLC. Alternatively, for a given memory capacity and *PE*, the probability $\Phi _\text {Ratio}$ of a ratio-based MLC will be significantly lower than $\Phi _\text {Re}$ in a resistance-based MLC: If $\Phi _\text {Re}=10^{-x}$, then $\Phi _\text {Ratio}$ is guaranteed to be between $10^{-\sqrt{2}x}$ and $10^{-2x}$ for a MLC system based on the same devices. To determine the effect of the *PE*, we fabricated an array of 1T1R hafnium-oxide devices and measured the effective variability of the encoded resistance states as a function of the number of programming iterations. We observed that a ratio-based MLC can reduce the programming time by 10$\times$, compared to a resistance-based MLC, under the same *n* and BEP.

## Assumptions and memory state encoding definition

We establish the following assumptions for resistance-switching devices used for both ReBE and RatioBE:

1. The resistance of a device can be set to any value within the range $[R_\text {on}, R_\text {off}]$ in which $R_\text {on}$ is the *nominal lowest resistance*, and $R_\text {off}$ the *nominal highest resistance*. Due to device variations, the resistance of a device may fall outside this nominal range.
2. The devices are programmed through an iterative write-verify mechanism, therefore their state can be set to any targeted value between $R_\text {on}$ and $R_\text {off}$ and within any specified tolerance range of the target value.
3. The *ON/OFF ratio* of the device, defined as $\alpha = R_\text {off}/R_\text {on}$, is a direct measure of the dynamic range of the state of the device.

For ReBE MLC memories, the state of a memory cell is defined as the electrical resistance of a single device, regardless of the mechanism used to retrieve its state (e.g. by applying a voltage across the device and sensing the resulting current, or by letting a current flow through the device and sensing the resulting voltage across it).

A RatioBE MLC uses two of such devices, namely $R_1$ and $R_2$, configured as a voltage divider to encode information (Fig. 1b). We define the *normalized state* *s* of the memory cell as the normalized output of the voltage divider:

1 $$\begin{aligned} s = \frac{V_\text {state}}{V_\text {read}} = \frac{R_2}{R_1+R_2} \end{aligned}$$

which is bound to the range (0, 1). Assuming that the resistance of $R_1$ and $R_2$ can be programmed to any value in the range $[R_\text {on}, R_\text {off}]$, we propose two encoding scenarios. In the first, we permanently fix one of the devices’ resistance, say $R_2$, and program $R_1$ to values in $[R_\text {on}, R_\text {off}]$. Under such a scenario, the maximum difference between the minimum and maximum memory state values (i.e. the maximum dynamic range) is $\tfrac{\sqrt{\alpha }-1}{\sqrt{\alpha }+1}$, which occurs when $R_2$ is set at $R_\text {on}\sqrt{\alpha }$. If we allow both devices to be dynamically programmed (the second type of encoding), we can potentially double the dynamic range, while the exact improvement depends on the ON/OFF ratio $\alpha$. To analyze the dynamic range under this encoding scenario, we split the range of memory states in two halves. In the first half, $R_2$ is fixed to $R_\text {on}$ and $R_1$ is programmed to values in $[R_\text {off}, R_\text {on}]$, resulting in an output in the range $(\tfrac{1}{\alpha +1},0.5)$. In the second half, $R_1$ is fixed to $R_\text {on}$ and $R_2$ is programmed to values in $[R_\text {on}, R_\text {off}]$, with an output falling within the range $(0.5, \tfrac{\alpha }{\alpha +1})$. Thus, the minimum and maximum state values for this second type of encoding would be $\tfrac{1}{\alpha +1}$ and $\tfrac{\alpha }{\alpha +1}$, respectively, resulting in a dynamic range of $\tfrac{\alpha -1}{\alpha +1}$. Note that the ratio between the dynamic ranges of the second and first encodings is $1+\tfrac{2\sqrt{\alpha }}{1+\alpha }$, revealing that the dynamic range of the second type of encoding is always greater (up to 2$\times$) than that of the first type. For a small ON/OFF ratio, the range increase is large (e.g. 1.75$\times$ for $\alpha =5$). For larger ON/OFF ratios, the increase is smaller (1.28$\times$ for $\alpha =50$, and 1.09$\times$ for $\alpha =500$). For the rest of our study, we focus on the second type of encoding, nevertheless, we note that whereas the first type results in a smaller dynamic range, it only programs a single device, instead of two, per memory write and thus incurs lower *PE*, which could be a preferred choice for devices with a greater ON/OFF ratio (e.g. > 500) where the benefit of increased dynamic range achieved by the second type of encoding becomes marginal.

## Bit error probability of multi-level memory cells

To design an *n*-level memory cell, either using ReBE or RatioBE, the choices of the *n* memory states and their corresponding $n-1$ decision thresholds for differentiating neighboring states are crucial for minimizing the BEP of the memory cell. In the following, we assume that after programming of a device to a targeted resistance, the probability distribution of the resulting resistance of the device is log-normally distributed and described by its mean $\mu = \log \mu ^*$ and standard deviation $\sigma = \log \sigma ^*$, where $\mu ^*$ and $\sigma ^*$ are their geometric or multiplicative mean and standard deviation, respectively.

Under the traditional ReBE, each state $s_i$ for $i\in \{1\cdots n\}$ is solely determined by the resistance of the device itself, thus the states can be characterized by the pairs $(\mu _i^*, \sigma _i^*)$, as shown in Fig. 2a. Being log-normally distributed, each state $s_i$ is centered on $\mu _i^*$ (not $\mu _i$). While $\mu _i^*$ has units of electrical resistance, $\sigma _i^*$ is a dimensionless multiplicative factor that measures the spread of the distribution of the state.

We further define $t_j$ for $j\in \{1\cdots n-1\}$ as the resistance threshold between states $s_j$ and $s_{j+1}$, therefore, $\mu _j^*<t_j<\mu _{j+1}^*$ (see Fig. 2a). By design, the first state $s_1$ is set to the lowest resistance $R_\text {on}$ while the last state $s_n$ is set to the highest resistance $R_\text {off}$, that is, $\mu _1^* = \mu _\text {on}^* = R_\text {on}$ and $\mu _n^* = \mu _\text {off}^* = R_\text {off}$.

Under the proposed RatioBE, where each state $s_i$ is encoded in a $R_1|R_2$ configuration using Eq. (1), $R_1$ and $R_2$ still follow the same log-normal distributions as of the ReBE, albeit with potentially different targeted resistance values. To avoid confusion, the geometric mean and geometric standard deviation of the resistance values used in this encoding are now denoted by $\nu _i^*$ and $\varsigma _i^*$, respectively. The distribution of each state $s_i$ and normalized thresholds $\tau _i$ are illustrated in Fig. 2b. Due to the symmetry of the encoding, for every state $\nu _i^*|\mu _\text {on}^*$ in the first half of the state range, there is a mirror state $\mu _\text {on}^*|\nu _i^*$ in the second half. Similarly, each normalized threshold in the second half $\tau _i'$ corresponds to $1-\tau _i$, where $\tau _i$ is the *i*-th threshold in the first half. If *n* is odd, there will be a state $s_{(n+1)/2}$ centered at $\tfrac{1}{2}$ with the pair $\mu _\text {on}^*|\mu _\text {on}^*$, as shown with a dashed curve in Fig. 2b. This state is not present if *n* is even. By design, the first and last states ($s_1$ and $s_n$) are given by $\mu _\text {off}^*|\mu _\text {on}^*$ and $\mu _\text {on}^*|\mu _\text {off}^*$, respectively.

Taking Fig. 2 as reference, if all *n* memory levels have the same probability of occurring, then the probability of encoding an error in a memory cell (BEP) can be expressed as:

2 $$\begin{aligned} \Phi = \frac{1}{n} \left[ \sum _{i=2}^n F_i(T_{i-1}) + \sum _{i=1}^{n-1}\left( 1-F_i(T_{i})\right) \right] \end{aligned}$$

where $T_i$ is the *i*-th decision threshold (either $t_i$ or $\tau _i$), and $F_i(T)$ is the cumulative distribution function (CDF) of state $s_i$ evaluated at threshold *T*. That is, $F_i(T)$ is the probability that state $s_i$ will be less than or equal to *T*, whereas, $1-F_i(T)$ is the probability that $s_i$ will be greater than *T*. Details on how to compute $F_i$ for ReBE and RatioBE are given in Supplementary Note 1. We use the BEP instead of more common reliability metrics, such as the bit error rate (BER), as the former exclusively involves errors due to the encoding itself, whereas the latter depends on the encoding and on additional aspects of the memory, such as the number of memory accesses per unit of time, the read/write ratio, and the read and supply voltages, among others.

The task is then to find the parameters that minimize function (2) for RatioBE ($\Phi _\text {Ratio}$) and ReBE ($\Phi _\text {Re}$). These parameters can be roughly split into two categories. The first category includes the physical device properties, such as the $R_\text {on}$ and $R_\text {off}$ values, the ON/OFF ratio $\alpha$, and the intrinsic variability of the devices (characterized by $\sigma ^*_i$ and $\varsigma ^*_i$). The second category includes architectural design parameters, such as the number of levels *n*, the mean resistance values for the states ($\mu ^*_i$ and $\nu ^*_i$), and decision thresholds ($t_i$ and $\tau _i$). Given a set of physical device parameters, Supplementary Table 1 summarizes the total number of architectural design parameters for an *n*-level memory. To enable thorough analysis and fair comparison, we assume that a device’s resistance after programming has an identical standard deviation across all targeted resistance values, thus $\sigma _i^* = \varsigma _i^* = \sigma ^*$. Having an identical standard deviation for different resistance states is a desired property of any resistance-based memory technology to be practically applicable, thus making this a valid assumption.

If the distribution of the device’s resistance follows a log-normal distribution with identical standard deviation, then function (2) is minimized when parameters $\mu _i^*$ and $t_i$ (for $\Phi _\text {Re}$) and $\nu _i^*$ and $\tau _i$ (for $\Phi _\text {Ratio}$), are such that the probability of encoding an error is identical for every state $s_i$, as we mathematically prove in Supplementary Note 2. The optimal parameters for ReBE and RatioBE are given in Supplementary Note 3. Substituting these optimal parameters into Eq. (2) (see Supplementary Note 4 for details) results in:

3 $$\begin{aligned} \Phi _\text {Re}&= \frac{n-1}{n}\hbox {erfc}\left[ \frac{\log \alpha }{2\sqrt{2}(n-1)\log \sigma ^*} \right] \end{aligned}$$

4 $$\begin{aligned} \Phi _\text {Ratio}&= \frac{n-1}{n}\hbox {erfc}\left[ \frac{\log \alpha }{2(n-1)\log \sigma ^*} \right] \end{aligned}$$

where $\hbox {erfc}$ is the complementary error function^29^.

## Discussion and comparison

Equations (3) and (4) are almost identical except for an additional $\sqrt{2}$ factor in the denominator of the error function in $\Phi _\text {Re}$. This factor can actually be intuitively explained through a simple mathematical transformation described as follows. Recall that for a ratio-based MLC, a state *s* depends on $R_1$ and $R_2$ as $s=R_2/(R_1+R_2)=1/(1+R_1/R_2)$. Let us consider the transformation $x\rightarrow 1/(1+x)$ where *x* is $R_1/R_2$. If the probability distribution of $R_1$ and $R_2$ are independent log-normal distributions with the same variance $\sigma$, then it can be proved that $R_1/R_2$ is log-normally distributed with variance $\sqrt{2}\sigma$. On the other hand, if the dynamic range of $R_i$ is $\alpha =R_\text {off}/R_\text {on}$ (for both $i=$ 1 and 2) then the dynamic range of $R_1/R_2$ would be $\alpha ^2$. Since the distribution of *x* is log-normal, which is exactly the same as that for ReBE except using new $\sigma '=\sqrt{2}\sigma$ and $\alpha '=\alpha ^2$, following the transformation $s=1/(1+x)$ we can deduce Eq. (4) from Eq. (3). Note that $\Phi _\text {Ratio}$ is proportional to $\hbox {erfc}(\tfrac{\log \alpha '}{\sigma '}) = \hbox {erfc}(\tfrac{\sqrt{2}\log \alpha }{\sigma })$, which is $\sqrt{2}$ times larger than the argument of the error function of $\Phi _\text {Re}$.

Equations (3) and (4) offer insights into the relationship between our figures of merit: BEP ($\Phi$), memory capacity (*n*) and programming effort (*PE* as a function of $\sigma ^*$). In particular, taking Fig. 3 as reference, we observe that for a target memory configuration (i.e. for given *n*, $\alpha$ and $\sigma ^*$) then:

1. The additional $\sqrt{2}$ factor in $\Phi _\text {Re}$ results in a significant lower $\Phi _\text {Ratio}$ compared to $\Phi _\text {Re}$ (check $\hbox {erfc}(x)$ v. $\hbox {erfc}(\sqrt{2}x)$ in Fig. 3a). In fact, deriving the ratio $\Phi _\text {Re}/\Phi _\text {Ratio}$ reveals that $\Phi _\text {Ratio} = \Phi _\text {Re}^\beta$ where $\beta$ is within $(\sqrt{2},2)$. In practice, $\beta$ will be closer to 2 than to $\sqrt{2}$. For instance, for $\Phi _\text {Re}$ being $10^{-3}$, $\Phi _\text {Ratio}$ would be less than $10^{-5}$ and thus $\beta >1.8$, whereas for $\Phi _\text {Re}=10^{-8}$ (achieved, for example, by increasing the *PE*), $\Phi _\text {Ratio}$ will be less than $10^{-15}$ and thus $\beta >1.9$.
2. For a given BEP, a memory cell using RatioBE can store $\sim$20–40% more bits compared to a memory cell using ReBE. For instance, in Fig. 3b–d, $\Phi _\text {Re}$ with $n=3$ (yellow, dashed line) is approximately the same as $\Phi _\text {Ratio}$ with $n=4$ (green, solid line), which represents a 33% increase in the number of bits per cell.
3. To achieve the same BEP as ReBE, RatioBE can allow higher device variability $\sigma ^{*\sqrt{2}}$, instead of $\sigma ^*$ for ReBE, and thus effectively reduce the *PE*. Note that even if the increase in $\sigma ^*$ is small (e.g. from 5 to 7%, or from 10 to 14%), the reduction in *PE* could be significant as the difficulty, time- and energy-wise, for reducing a device’s effective variability increases as we increment the number of device programming iterations^28^.
4. For both encoding schemes, changing the ON/OFF ratio from $\alpha$ to $\alpha ^k$ (e.g. by using a different device) produces a similar effect on the BEP as modifying the number of memory levels from *n* to $1+(n-1)/k$, while keeping the same *PE* (i.e. fixed $\sigma ^*$). This means if we increase $\alpha$ from 10 to $10^2$ (or $10^3$) while fixing the *PE*, we can increase *n* from 2 to 3 (or 4) without affecting the BEP (see grey bars in Fig. 3b). Alternatively, with a fixed *n*, the change $\alpha \rightarrow \alpha ^k$ has equivalent effect on BEP as the change of device’s variability $\sigma ^*\rightarrow \root k \of {\sigma ^*}$. For instance, for $n=8$ and $\sigma ^*=10\%$ in Fig. 3c, changing $\alpha$ from 10 to 100 (i.e. $k=2$) results in the same reduction as that caused by decreasing $\sigma ^*$ from 10 to 5% (because $\sqrt{1.10}\approx 1.05$) when keeping $\alpha = 10$ (Fig. 3d).
5. Even if the developed model assumes uniform resistance-based distributions across the resistance range, we can delimit the BEP of a non-uniform state distribution within a range using two uniform resistance distributions (as shown in Supplementary Figure 1), for which we can fully describe with the proposed model.

**Figure 4:**
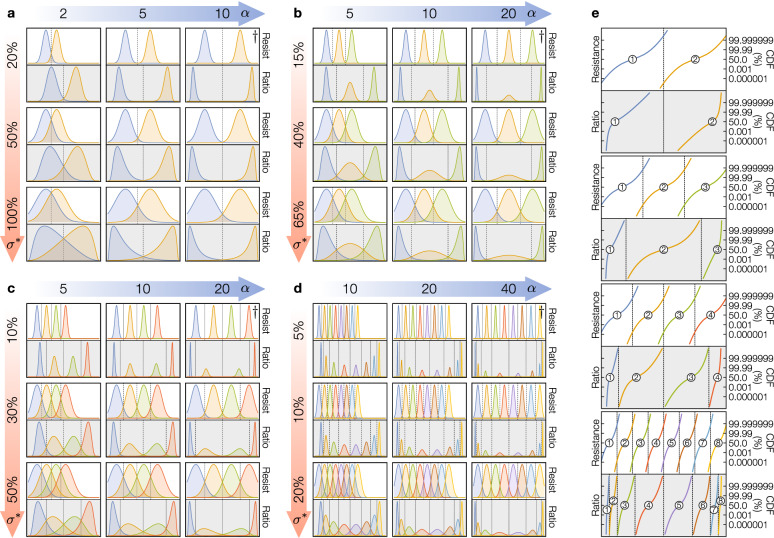
Multi-level state distribution under different parameters. (**a**) Two-level cell with $\alpha$ in [2, 10] and $\sigma ^*$ in $[20\%,100\%]$. (**b**) Three-level cell with $\alpha$ in [5, 20] and $\sigma ^*$ in $[15\%,65\%]$. (**c**) Four-level cell with $\alpha$ in [5, 20] and $\sigma ^*$ in $[10\%,50\%]$. (**d**) Eight-level cell with $\alpha$ in [10, 40] and $\sigma ^*$ in $[5\%,20\%]$. **e**, Cumulative distribution function (CDF) of the states for configurations marked with a $\dagger$ in (**a**–**d**) (in the right-top corners). In all cases, the optimal thresholds between states are marked with dashed vertical lines, and for a given configuration of *n*, $\alpha$ and $\sigma ^*$, the top part uses ReBE and the bottom part RatioBE (on a grey background).

**Figure 5:**
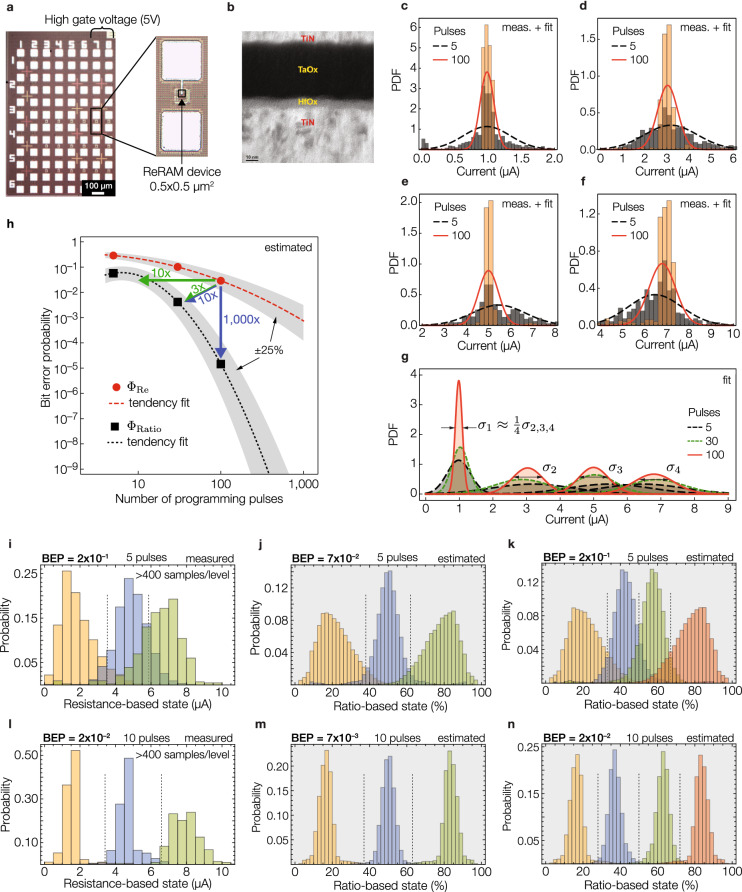
1T1R hafnium-oxide devices and their state distribution. (**a**) Microscope image of a section of the 1 Kb array of 1T1R hafnium-oxide devices (scale bar, 100 $\upmu$m), and a detail of one ReRAM device. (**b**) TEM image of the TiN/TaOx/HfOx/TiN stack for the ReRAM devices (scale bar, 10 nm). (**c–f**) Histograms and normal fits for states centered at 1, 3, 5 and 7 $\upmu \hbox {A}$ (read voltage of 100 mV), respectively, using 5 and 100 programming pulses. Each histogram uses a sample size of at least 480 programming cycles. (**g**) Probability density function fits of the four states, using 5, 30 and 100 programming pulses. (**h**) Estimated BEP for ReBE (in red) and RatioBE (in black). In both cases, as a visual aid, we included a tendency fit with dashed lines and an error band of ±25% was added on the estimation of the BEP to due the uncertainty introduced on the modeling of the distributions (normal vs. log-normal). (**i–n**) Second experiment using the same 1T1R array but from a different batch. Figures (**i**) and (**l**) were experimentally obtained, whereas Figures (**j**,**k**,**m**,**n**) were estimated based on the experimental data measured for figures (**i**) and (**l**).

**Figure 6:**
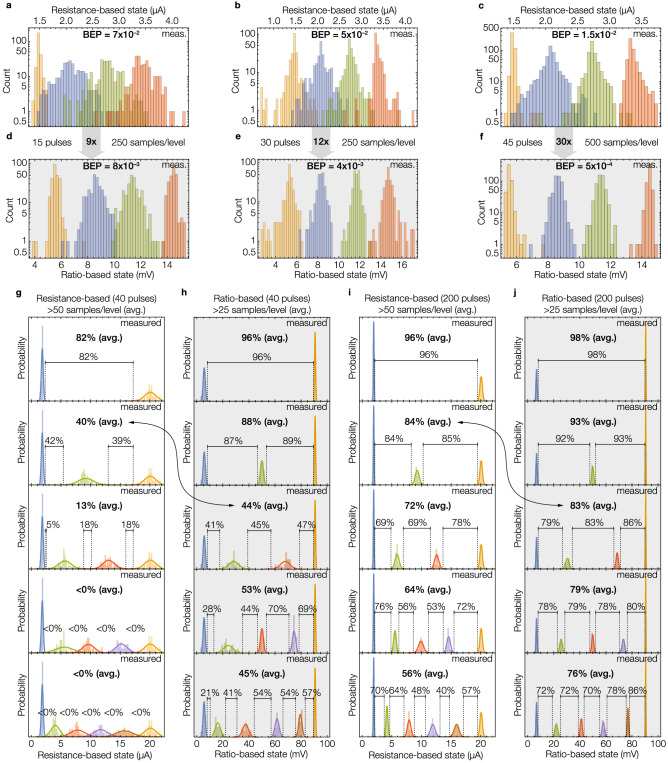
Additional experimental evaluation. (**a–f**) Multilevel programming on tungsten-based commercially available devices from Knowm, using a small ON/OFF ratio of $\alpha =2.2$. (**g–j**) Multilevel programming of 2–6 levels using higher-resolution titanium-based devices. Instead of the BEP previously used, here we employed the size of the window between adjacent states to compare the reliability between resistance-based and ratio-based encodings.

To visualize the effects of *n*, $\alpha$ and $\sigma ^*$, Fig. 4a–d illustrate the memory state distributions and locations of the optimal thresholds $t_i$ and $\tau _i$ as a function of $\alpha$ and $\sigma ^*$ for 2–8 levels. Note that even with identical device variances across its resistance range, the ratio-based states are not equally distributed, with wider distributions towards the center of the ratio-based state range. While the optimal decision thresholds $t_i$ for ReBE are logarithmically uniformly distributed, the ratio-based thresholds $\tau _i$ are not. The degree of this non-uniformity is a function of $\alpha$. For a smaller ON/OFF ratio (e.g. $\alpha =5$) the ratio-based states are compressed towards the center and more similar to each other, whereas for a higher ratio (e.g. $\alpha =20$) the distributions of the first and last states are sharper, compared to the ones near the center. As a reference, Fig. 4e compares the resistance-based and ratio-based CDFs that we could expect if we implemented a MLC memory with the four configurations marked with a $\dagger$ (i.e. the right-top configurations) in Fig. 4a–d. Note that, for the shown scale (BEP at $10^{-8}$), there is an overlap between states for ReBE, not present for RatioBE.

## Experimental evaluation

To validate the proposed multi-level encoding mechanism, we experimentally compared the BEP performance of RatioBE and ReBE under various parameter settings, including number of levels, ON/OFF ratio $\alpha$, and programming effort *PE*, based on three different types of devices: a 1T1R hafnium-based array, a set of commercially available tungsten-based devices, and a set of titanium-based devices.

### 1T1R array

To estimate the effect of the *PE* on the effective variability of the resistive devices, we conducted experiments on a 1 Kb 1T1R array (Fig. 5a) of TiN/TaOx/HfOx/TiN (Fig. 5b) ReRAM devices (see “Methods” for more details). A train of pulses with different number of pulses was applied to program the devices at four levels centered at 1, 3, 5 and 7 $\upmu \hbox {A}$ (read voltage of 100 mV). The distribution of these four states was used to estimate the BEP that we would obtain under the RatioBE and ReBE with these devices as a function of the number of programming pulses. Figure 5c–f show the histograms and truncated normal fits (to avoid negative conductance) for each state using 5 and 100 programming pulses. Figure 5g summarizes the distribution of the four states using 5, 30 and 100 pulses. The bit error probabilities $\Phi _\text {Re}$ and $\Phi _\text {Ratio}$ were computed based on Eq. (2) using the parameters listed in Supplementary Table 2 and Supplementary Note 5. Figure 5h shows $\Phi _\text {Re}$ and $\Phi _\text {Ratio}$ as a function of the number of programming pulses. Note that RatioBE can achieve 1000$\times$ reduction in BEP, compared to ReBE. Alternatively, we can reduce the *PE* by a factor of 10 (from 100 to 10 programming pulses) thus significantly decrease the programming time and programming energy while achieving similar BEP. Also note that while the experimental data was modeled assuming normally distributed current-based states, the experimental results are congruent with our log-normally resistance-based modeling (compare Figs. 3b–d with 5h) from which we observe that varying the ON/OFF ratio $\alpha$ has roughly a similar effect on BEP as varying the number of programming pulses. We used normal distributions, as opposed to log-normal distributions, to model the current-based states as it is commonly done in the literature^30,31^. We found that the estimation of the BEP assuming normally distributed states was within ±25% of the estimated BEP assuming log-normally distributed states. As a visual aid, Fig. 5h includes such an error band of ±25%. Supplementary Figure 2 visually compares the effect of a normal fit with a log-normal fit of the four current-based states. Supplementary Figure 3 shows that the error introduced on the BEP due to the truncation of the normally distributed states is less than 7%. In Fig. 5i–n we performed additional experiments on the same 1T1R array but from a different fabrication batch. We compare the BER of a 3-level ReBE (Fig. 5i) with a 3- and a 4-level RatioBE (Fig. 5j,k, respectively) using 5 and 10 programming pulses (Fig. 5l–n), respectively. In accordance to the mathematical model, we observed that $\Phi _\text {Ratio}$ of a 4-level cell is comparable to $\Phi _\text {Re}$ of a 3-level cell (compare Fig. 5k vs. Fig. 5i and Fig. 5n vs. Fig. 5l).

### Commercially available devices

We also compared both encodings using tungsten-based devices from Knowm^32^. Figure 6a–f summarize the results obtained based on these devices using a smaller ON/OFF ratio $\alpha =2.2$ (compared to $\alpha =7$ in the 1T1R array) and under different numbers of programming pulses (15, 30 and 45). Instead of modeling the distributions of the states to estimate the BEP (as done with the 1T1R array), we report the BEP by directly counting the number of encoding errors. We observed the same trend as before in which higher *PE* (i.e. more programming pulses) results in not only narrower state distributions for both encodings, but also that the relative reduction of $\Phi _\text {Ratio}$ with respect to $\Phi _\text {Re}$ is greater as the *PE* increases (see that the increase from 9$\times$ to 30$\times$ in Fig. 6a–f is consistent with the trend shown in Fig. 3).

### Titanium-based devices

Finally, to evaluate MLC memories with more levels and under different *PE*, we compared the encodings using a multi-level titanium-based device^33^. Figure 6g–j show the experimental results for 2–6 levels, and with 40 and 200 programming pulses (Fig. 6g–j, respectively). Since the state distributions were significantly sharper, the number of encoding errors was zero for most cases under the limited test time. Instead of using BEP as the evaluation metric, we report the size of the normalized *eye window* or *margin* between adjacent states, which is also a direct, and commonly used, measure of the reliability of the memory encoding. Rather than mapping a given margin to a BEP value, we used the data to compare the reliability among different cases. Consistent with Fig. 5h for the 1T1R array, we found that a RatioBE can yield about the same reliability as a ReBE, while using only 20% (40 vs. 200 programming pulses) of the time and energy to program the devices (observe the similar margins in Fig. 6h,i). The similar BEP between a 3-level ReBE and a 4-level RatioBE predicted by our aforementioned model was also observed in these devices (compare their similar margins indicated by the two-sided arrow between Fig. 6g and h, and between Fig. 6i and j).

## Concluding remarks

We present a general mechanism to encode multi-bit information on resistance-switching devices that results in significant improvement to the bit error probability (BEP), and/or drastic reduction of the energy or time needed for programming the devices. The proposed ratio-based encoding (RatioBE) uses the resistance ratio of two devices, as opposed to the resistance of a single device employed in the traditional resistance-based encoding (ReBE), to store information. Our experimental data on multiple types of ReRAM devices suggests that, compared to ReBE, our RatioBE can offer (1) a reduction of 5–10$\times$ in the programming time and energy while maintaining the same BEP, or (2) a reduction of up to 1000$\times$ in the BEP while consuming the same programming time/energy, or (3) a combination of both: 3$\times$ time-energy reduction and 10$\times$ BEP reduction. Note that this encoding mechanism should not be exclusive to ReRAM devices; it could also be applicable to other resistance-switching devices, such as phase change memory (PCM).

To gain insights into the fundamental advantages of RatioBE over ReBE, we derived a closed-form expression to estimate the BEP for both encodings. Assuming log-normally distributed resistances on the states of the devices, we found that the main reason why RatioBE is superior to ReBE is that the argument in the error function of the RatioBE expression is $\sqrt{2}$ times larger than that for ReBE. For practical applications, this factor means that for a multi-level resistance-based memory cell, such as a 1T1R cell, with a BEP of $10^{-x}$, we can construct a ratio-based 1T2R memory cell with similar memory footprint (since the transistor typically dominates the size of the cell) but with a BEP that is close to $10^{-2x}$. Alternatively, if we fix the BEP, we could increase the number of bits per cell by 20–40%, or significantly reduce the time and/or energy employed to program the devices. Whereas the actual reduction in programming time or energy would be device-dependent, and thus harder to generalize, the observed 5–10$\times$ reduction in our experimental data demonstrate that the proposed idea works and is promising. We believe that this type of multi-level RatioBE will be essential to tackle the intrinsic variability of ReRAM and other resistance-switching devices, and thus an important enabler for the next generation of high-performance and high-capacity non-volatile memories.

## Methods

The ReRAM devices (Fig. 5a) were integrated on top of an array of transistors using the transistor’s drain as contact point. The array contains 128 rows and 8 columns, from which we used the high-voltage (5 V) columns 7 and 8. The transistor, which is fabricated in a 130 nm CMOS technology node, is designed to accurately control the compliance current of the ReRAM devices. The ReRAM device under test is a TiN/TaOx/HfOx/TiN stack (Fig. 5b). The HfOx is a switching layer, and the oxygen-deficient TaOx is a capping layer to store oxygen and modulate the local temperature during the resistive switching process. Based on these high performance devices, a series of exploratory experiments were developed to implement 3 or 4 resistance levels with a customized array-test platform. For each level, a train of pulses with different number of pulses were applied with fixed pulse width (50 ns) and fixed amplitude to obtain a relatively tight conductance distribution. The same train was applied at least 48 times to 5 different devices during a RESET operation, and another similar set of pulses during a SET operation, for a total of 480 programming cycles. For different levels, amplitudes of drain voltage in SET operation varied from 1.3 to 3.0 V with gate voltage from 1.6 to 2.0 V. The amplitudes of source voltage in RESET operation varied from 1.5 to 3.0 V with gate voltage at 5.0 V. We defined four levels centered at 1, 3, 5 and 7 $\upmu \hbox {A}$ with a read voltage of 100 mV. Since the states were uniformly distributed in current (not in resistance), each state was modeled based on the conductance of the device and as a normal distribution whose variance depends on the number of programming pulses. The employed programming mechanism produced nonuniform variances on the states’ conductances, particularly for the first state (centered at 1 $\upmu \hbox {A}$) which had a standard deviation of (on average) 4 times smaller than that of the other three levels. As expected, for all cases, we observed a clear reduction in the variance of each state as we increased the number of programming pulses (see Supplementary Table 2 for details). We used these measurement data to predict and compare the BEP in a 4-level resistance-based and ratio-based MLCs. Whereas in the former we directly encoded information on the four levels that we experimentally measured (1, 3, 5 and 7 $\upmu \hbox {A}$), in the latter we had to interpolate the dataset to estimate the variance that a device would have if programmed to a value other than the ones we measured. Supplementary Note 5 provides details on the parameters used for each state in both MLCs. For the second experiment on the 1T1R devices, due to a limitation on the test equipment, while performing a RatioBE write, we could not simultaneously measure the output of the voltage divider while programming the devices. Instead, we measured the individual resistance of the devices $R_1$ and $R_2$ using and predicted the normalized ratio-based state using Eq. (1). We measured the quality of estimating the output voltage by measuring the individual resistances in Supplementary Figure 4 using the titanium-based devices, in which we compared the estimated normalized state with the actual measurement of the voltage divider. We found a very high correlation between both experiments (Pearson correlation coefficient of 0.999). For the tungsten-based and we employed a similar iterative programming algorithm, but we were able to simultaneously measure the resistance-based and ratio-based states. We used a read voltage of 20 mV and centered the resistance-based states at 1.5, 2.1, 2.7 and 3.3 $\upmu \hbox {A}$. The intermediate resistance-based state used for the ratio-based encoding was centered at 2.4 $\upmu \hbox {A}$. For the titanium-based devices we used the same programming algorithm as with tungsten-based devices, but with a read voltage of 100 mV and a current range from 2 to 20 $\upmu \hbox {A}$.

## Supplementary information

Supplementary Information

## Data Availability

The data that support the plots within this paper and other findings of this study are available from the corresponding authors upon reasonable request.
