# Supplementary material for: Ratio-based multi-level resistive memory cells
Source: Sci Rep. 2021 Jan 14;11:1351. doi: 10.1038/s41598-020-80121-7 (PMC7809403; doi:10.1038/s41598-020-80121-7)
Supplement: Supplementary file 1 — Supplementary Information [file 41598_2020_80121_MOESM1_ESM.pdf]

Ratio-based Multi-level Resistive Memory Cells

Miguel Angel Lastras Montaño[*a], Osvaldo Del Pozo Zamudio[a], Lev Glebsky[a], Meiran Zhao[b], Huaqiang Wu[b], and Kwang-Ting Cheng[†c]

[a] *Instituto de Investigación en Comunicación Óptica, Facultad de Ciencias, Universidad Autónoma de San Luis Potosí, México*
[b] *Institute of Microelectronics, Tsinghua University, Beijing, China*
[c] *School of Engineering, Hong Kong University of Science and Technology, Clear Water Bay, Kowloon, Hong Kong*

Supplementary Information

[*]Corresponding author (miguel.lastras@uaslp.mx)
[†]Corresponding author (timcheng@ust.hk)

Supplementary Note 1: Computing the CDF for a resistance- and ratio-based encoding

To compute the cumulative distribution function (CDF) $F_i(T)$ of state s_i evaluated at threshold T, we generally need the integral of the probability density function (PDF) f_i. In a resistance-based encoding with log-normally distributed states described by the pairs (μ_i, σ_i), the function $f_{i,\text{res}}$ is the PDF of a log-normal random variable:

$$f_{i,\text{res}}(x) = \frac{1}{\sqrt{2\pi}x\sigma_i}\exp\left[-\frac{(\log x - \mu_i)^2}{2\sigma_i^2}\right], \tag{SM.1.1}$$

therefore

$$F_{i,\text{res}}(t_i) = \int_0^{t_i} f_{i,\text{res}}(x)\,\mathrm{d}x = \frac{1}{2}\operatorname{erfc}\left[\frac{\mu_i - \log t_i}{\sqrt{2}\sigma_i}\right] \tag{SM.1.2}$$

where erfc is the complementary error function, defined as $\operatorname{erfc} x = 1 - \operatorname{erf} x$, and erf is the error function. In a ratio-based encoding, however, the states are given by the transformation

$$s_i = \frac{Y_i}{X_i + Y_i} = \frac{1}{1 + \frac{X_i}{Y_i}} \tag{SM.1.3}$$

where X_i and Y_i are independent log-normal variables (each following equation (SM.1.1)) described by the pairs (ν_X, ς_X) and (ν_Y, ς_Y), respectively. This results in log-normal distribution for X/Y with log-variance $\sqrt{\varsigma_X^2 + \varsigma_Y^2}$ and median $\nu_X - \nu_Y$. In order to obtain a distribution function for ratio-based state s_i we apply transformation $x \to 1/(1+x)$ for the log-normal distribution of X/Y. As a result we get the PDF $f_{i,\text{rat}}(x)$ for ratio-based state s_i:

$$f_{i,\text{rat}}(x) = \frac{1}{\sqrt{2\pi}x(1-x)\sqrt{\varsigma_{iX}^2 + \varsigma_{iY}^2}}\exp\left[-\frac{\left(\log\frac{x}{1-x} + \nu_{iX} - \nu_{iY}\right)^2}{2(\varsigma_{iX}^2 + \varsigma_{iY}^2)}\right]. \tag{SM.1.4}$$

where x runs over $[0, 1]$, i.e. the range of s_i. Whereas equation (SM.1.4) can be used to plot the shape of the ratio-based states, the expression is harder to integrate. Instead, to find $F_{i,\text{res}}(\tau_i)$, we compute the joint probability of X_{iX} and X_{iY} transformed by equation (SM.1.3). For this, we note that

$$F_{i,\text{res}}(\tau_i) = \Pr\left[\frac{Y_i}{X_i + Y_i} \le \tau_i\right] = \Pr[Y_i \le X_i \cdot \phi_i]$$

where $\phi_i = \tau_i/(1-\tau_i)$. Therefore, assuming that X_i and Y_i are independent and with identical variances (i.e. $\varsigma_{iX} = \varsigma_{iY} = \sigma$), then

$$\begin{aligned} F_{i,\text{res}}(\tau_i) = \iint\limits_{Y_i \le X_i\cdot\phi_i} f_{i,\text{res}}(x) f_{i,\text{res}}(y)\,\mathrm{d}x\,\mathrm{d}y &= \int_0^\infty f_{i,\text{res}}(y)\left[\int_0^{y\phi_i} f_{i,\text{res}}(x)\,\mathrm{d}x\right]\mathrm{d}y \\ &= \frac{1}{2}\operatorname{erfc}\left[\frac{\log\left(\frac{1-\tau_i}{\tau_i}\right) + \nu_{iY} - \nu_{iX}}{2\sigma}\right] \end{aligned} \tag{SM.1.5}$$

Supplementary Note 2: Parameter optimality of resistance-based encoding

After proper change of variables in equation (2) in the main manuscript of the article, we get the problem of finding conditional minimum of the function

$$\Phi(\Delta) = C - \sum_{j=1}^{m} \phi(\Delta_j) \qquad (\bullet)$$

with the condition

$$\sum_{j=1}^{m} \Delta_j = \gamma, \qquad (\bullet\bullet)$$

where $\gamma > 0$ We may use the method of Lagrange multipliers, that gives as Lagrange function

$$\Lambda = \Phi(\Delta) + \lambda(\sum_{j=1}^{m} \Delta_j).$$

It gives us

$$\frac{\partial \Lambda}{\partial \Delta_i} = -\phi'(\Delta_i) + \lambda.$$

Using the fact that $\phi(x) = a\,\mathrm{erf}(bx)$ for some a and b we get

$$\exp(-b\Delta_i^2) = \frac{\lambda}{ab}.$$

Taking logarithm and introducing a new constant $c > 0$ (depending on a, b and λ) we get

$$|\Delta_i| = c.$$

If the signs of Δ_i are fixed, we may find c by the relation ($\bullet\bullet$):

- if all $\Delta_i > 0$, then $\Delta_i = \frac{\gamma}{m}$;
- if k of Δ_i are negative we get $|\Delta_i| = \frac{\gamma}{m-2k}$, ($m - 2k$ should be positive as $\gamma > 0$). It follows that $k = 0$ if $m \leq 2$.

Which extreme are minimal

Proposition 2.1. *Then the point $\Delta_i = \frac{\gamma}{m}$ is the only minim for ($\bullet$) with condition ($\bullet\bullet$). Moreover, $\Phi_{min} = C - m\phi(\frac{\gamma}{m})$.*

Proof. It suffices to check extremum found above. Recall, that existence of extrema with $\Delta_i < 0$ implies that $m \geq 3$. Let $H = \{\frac{\partial^2 \Phi}{\partial \Delta_i \partial \Delta_j}\}$. For extremal points $H = C\,\mathrm{diag}\{sign(\Delta_i)\}$, with constant $C > 0$. As our condition is lineal the extremum is minimum if and only if H is positive definite on subspace $\sum_{i=1}^{m} \epsilon_i = 0$.

1. if all $\Delta_i > 0$ the matrix H is positive definite and the extrema is minima.
2. if at least two Δ_i are negative then maximal positive subspaces of H has dimension less than $m - 2$. Our subspace is of dimension $m - 1$ and can not be positive.
3. if just one Δ_i is negative, say $\Delta_1 < 0$, there exists $\Delta_2 = \Delta_3 > 0$. Let $\epsilon = (1, -1/2, -1/2, 0, 0, \dots)$. Clearly ϵ belongs to our subspace and $\langle \epsilon, H\epsilon \rangle = -C/2 < 0$.

□

For the case of resistance-based memory we have $m = 2n - 2$, $\Delta_{2i-1} = \log t_i - \mu_i$ and $\Delta_{2i} = \mu_{i+1} - \log t_i$, for $i = 1, 2, \dots, n-1$. As we have explained the case of ratio-based memory may be reduced to the same calculation.

Supplementary Note 3: Optimal parameters

For ReBE, the optimal parameters are:

$$\begin{aligned} \mu_i^* &= R_{\text{on}} \cdot \alpha^{\frac{i-1}{n-1}} \\ t_i &= R_{\text{on}} \cdot \alpha^{\frac{2i-1}{2n-2}} \end{aligned} \tag{SM.3.1}$$

whereas for RatioBE, the optimal parameters are:

$$\begin{aligned} \nu_i^* &= R_{\text{on}} \cdot \alpha^{\frac{n+1-2i}{n-1}} \\ \tau_i &= \frac{1}{1+\alpha^{\frac{n-2i}{n-1}}} \end{aligned} \tag{SM.3.2}$$

Supplementary Note 4: Probability of encoding an error (closed form)

From equation (2) in the manuscript, the probability Φ_{Re} of erroneously encoding a bit in a resistance-based encoding is given by:

$$\begin{aligned}
\Phi_{\text{Re}} &= \frac{1}{n}\left[\sum_{i=2}^{n} F_{i,\text{res}}(t_{i-1}) + \sum_{i=1}^{n-1}\left(1 - F_{i,\text{res}}(t_i)\right)\right] \\
&= \frac{1}{n}\left[\sum_{i=2}^{n} \frac{1}{2}\operatorname{erfc}\left[\frac{\mu_i - \log t_{i-1}}{\sqrt{2}\sigma_i}\right] + \sum_{i=1}^{n-1}\left(1 - \frac{1}{2}\operatorname{erfc}\left[\frac{\mu_i - \log t_i}{\sqrt{2}\sigma_i}\right]\right)\right] \\
&= \frac{1}{n}\left[\sum_{i=2}^{n} \frac{1}{2}\operatorname{erfc}\left[\frac{\log(\mu_i^*/t_{i-1})}{\sqrt{2}\log\sigma_i^*}\right] + \sum_{i=1}^{n-1}\left(1 - \frac{1}{2}\operatorname{erfc}\left[\frac{\log(\mu_i^*/t_i)}{\sqrt{2}\log\sigma_i^*}\right]\right)\right].
\end{aligned}$$

If we substitute the optimal values for μ_i^* and t_i (from equation (3) in the manuscript) and assume uniform variances across all resistance values (i.e. $\sigma_i = \sigma$), we find that the all the terms in the sums are independent of i:

$$\begin{aligned}
\Phi_{\text{Re}} &= \frac{1}{2n}\left[\sum_{i=2}^{n} \operatorname{erfc}\left[\frac{\log\alpha}{2\sqrt{2}(n-1)\log\sigma^*}\right] + \sum_{i=1}^{n-1}\left(1 + \text{erf}\left[-\frac{\log\alpha}{2\sqrt{2}(n-1)\log\sigma^*}\right]\right)\right] \\
&= \frac{1}{2n}\left[\sum_{i=2}^{n} \operatorname{erfc}\left[\frac{\log\alpha}{2\sqrt{2}(n-1)\log\sigma^*}\right] + \sum_{i=1}^{n-1}\left(1 - \text{erf}\left[\frac{\log\alpha}{2\sqrt{2}(n-1)\log\sigma^*}\right]\right)\right] \\
&= \frac{1}{2n}\left[\sum_{i=2}^{n} \operatorname{erfc}\left[\frac{\log\alpha}{2\sqrt{2}(n-1)\log\sigma^*}\right] + \sum_{i=1}^{n-1} \operatorname{erfc}\left[\frac{\log\alpha}{2\sqrt{2}(n-1)\log\sigma^*}\right]\right] \\
&= \frac{n-1}{n}\operatorname{erfc}\left[\frac{\log\alpha}{2\sqrt{2}(n-1)\log\sigma^*}\right]. \qquad \text{(SM.4.1)}
\end{aligned}$$

Similarly, for a ratio-based encoding, assuming that $\varsigma_i = \sigma$ and recalling that for the first half of the states $\nu_{iY}^* = \mu_{\text{on}}^*$ and $\nu_{iX}^* = \nu_i^*$, the probability Φ_{Ratio} of erroneously encoding a bit is given by:

$$\begin{aligned}
\Phi_{\text{Ratio}} &= \frac{1}{n}\left[\sum_{i=2}^{n} F_{i,\text{rat}}(\tau_{i-1}) + \sum_{i=1}^{n-1}\left(1 - F_{i,\text{rat}}(\tau_i)\right)\right] \\
&= \frac{1}{n}\left[\sum_{i=2}^{n} \frac{1}{2}\operatorname{erfc}\left[\frac{\log\left(\frac{1-\tau_{i-1}}{\tau_{i-1}}\frac{\mu_{\text{on}}^*}{\nu_i^*}\right)}{2\log\sigma^*}\right] + \sum_{i=1}^{n-1}\left(1 - \frac{1}{2}\operatorname{erfc}\left[\frac{\log\left(\frac{1-\tau_i}{\tau_i}\frac{\mu_{\text{on}}^*}{\nu_i^*}\right)}{2\log\sigma^*}\right]\right)\right].
\end{aligned}$$

Now substituting the optimal values for ν_i^* and τ_i (from equation (4) in the manuscript), we again find that all the terms in the sums are independent of i:

$$\begin{aligned}
\Phi_{\text{Ratio}} &= \frac{1}{2n}\left[\sum_{i=2}^{n} \operatorname{erfc}\left[\frac{\log\alpha}{2(n-1)\log\sigma^*}\right] + \sum_{i=1}^{n-1}\left(1 - \text{erf}\left[\frac{\log\alpha}{2(n-1)\log\sigma^*}\right]\right)\right] \\
&= \frac{1}{2n}\left[\sum_{i=2}^{n} \operatorname{erfc}\left[\frac{\log\alpha}{2(n-1)\log\sigma^*}\right] + \sum_{i=1}^{n-1} \operatorname{erfc}\left[\frac{\log\alpha}{2(n-1)\log\sigma^*}\right]\right] \\
&= \frac{n-1}{n}\operatorname{erfc}\left[\frac{\log\alpha}{2(n-1)\log\sigma^*}\right]. \qquad \text{(SM.4.2)}
\end{aligned}$$

Supplementary Note 5: Experimental parameter extraction

For the 4-level resistance-based study case, we used the four levels that were experimentally measured (centered at 1, 3, 5 and 7 μA) to directly represent the four states of the memory. The experimental parameters were extracted by computing a truncated standard distribution (from 0 to ∞) from the experimental data of each state. The decision thresholds t_i between each state were computed as to minimize the average bit error probability for all four levels. Supplementary Table 2 shows the parameters used for the resistance-based encoding. Note that these states are proportional to the conductance of the devices. Therefore, in order to model a ratio-based encoding, we need to take the inverse of the states (since they are in units of current) to transform the states to resistances. Also note that the first state of the memory (centered at 1 μA) represents the higher resistance state R_{off}, whereas the last state (centered at 7 μA) represents the lowest resistance state R_{on}. These two resistive states are used to define the ratio-based states.

For the 4-level ratio-based study case, in addition to the R_{on} and R_{off} parameters in Supplementary Table 2, we need to determine one more intermediate state (centered at ν and given in units of current), its standard deviation (ς), and one decision threshold (τ). The configurations per state (given with a $R_1|R_2$) are the following:

- State 1: $R_{\text{off}}|R_{\text{on}}$
- State 2: $R_{\nu}|R_{\text{on}}$
- State 3: $R_{\text{on}}|R_{\nu}$
- State 4: $R_{\text{on}}|R_{\text{off}}$

where R_{ν} is the resistance of a device centered at ν μA. We determined ν and its standard deviation ς using the experimental measurements at 1, 3, 5 and 7 μA as follows: First, we estimated ς by interpolating the standard deviation σ in Table 2 as a function of the mean value μ. Then we searched for a value of ν such that the overall bit error probability of the four ratio-based levels is minimized. These parameters, given as the pair (ν, ς), are: $(4.1, 1.2)$, $(4.1, 0.71)$ and $(3.9, 0.453)$ for 5, 30 and 100 programming pulses, respectively. Similarly, we used the following decision thresholds τ: 0.23, 0.225 and 0.21 for 5, 30 and 100 programming pulses, respectively. As previously mentioned, the values for ν and ς are given in μA. The values for τ are unitless (recall that the ratio-based encoding uses a normalized state definition).

Supplementary Table 1: Number of design parameters for a MLC

Supplementary Table 1: Number of design parameters for an n-level memory cell. Note that σ_i^* and ς_i^* are physical parameters, (not design parameters), but are included in the table because they can be effectively modified by adjusting the *PE*.

	μ_i^*	t_i^*	σ_i^*	ν_i^*	τ_i^*	ς_i^*	total
Resistance-based	$n-2$	$n-1$	n	–	–	–	$3n-3$
Ratio-based (even n)	–	–	–	$(n-2)/2$	$(n-2)/2$	$(n+2)/2$	$(3n-2)/2$
Ratio-based (odd n)	–	–	–	$(n-3)/2$	$(n-1)/2$	$(n+1)/2$	$(3n-3)/2$

Supplementary Table 2: Experimental parameters for the resistance-based encoding

Supplementary Table 2: Mean (μ), standard deviation (σ), and the decision thresholds (t) used in the 4-level resistance-based study case, as a function of the number of programming pulses (5, 30 or 100). All the parameters are in μA.

	State 1		t_1	State 2		t_2	State 3		t_3	State 1	
Pulses	μ_1	σ_1		μ_2	σ_2		μ_3	σ_3		μ_4	σ_4
5	0.982	0.352	1.69	3.18	1.2	4.32	5.46	1.21	5.96	6.46	1.21
30	1.03	0.254	1.58	2.87	0.82	4.04	5.03	0.62	5.9	6.86	0.83
100	0.984	0.105	1.4	3.03	0.457	4.02	4.99	0.448	5.81	6.79	0.6

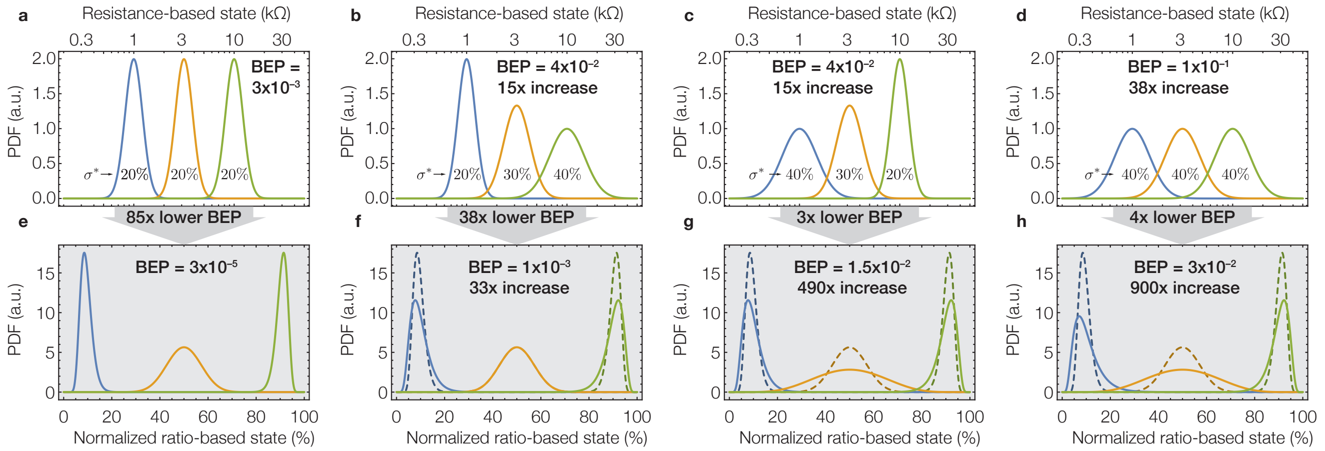

Supplementary Figure 1: **Effect of non-uniform resistance distribution on the BEP**. **a**. 3-level memory with uniform resistance distribution with $\sigma^* * = 20\%$ between 1 kΩ and 10 kΩ, producing a BEP of 3×10^{-3}. **b**. The same memory memory as in part **a**, but with increasingly wider higher resistance states (σ^* from 20% to 40%). **c**. The same memory memory as in part a, but with increasingly wider lower resistance states (σ^* from 20% to 40%). d. The same memory memory as in part a, but with wider uniform resistance states distribution ($\sigma^* = 40\%$). **e-h**. The ratio-based state distribution for configurations shown in **a-d**, respectively. Figures **b-d** show the relative increase in BEP with respect to the case in Figure **a**. Figures **f-h** show the relative increase in BEP with respect to the case in Figure **e**. As a visual add, in Figures **f-h** we include with dashed curves the state distributions of Figure **e**. Figure **g** shows a significantly higher increase on BEP compared to Figure **f** (490x vs. 33x), that is due to the fact that the middle ratio-based state (shown in yellow) is defined with two devices in the lowest resistance state, which is wider in Figure **g** compared to Figure **f** (40% vs. 20%).

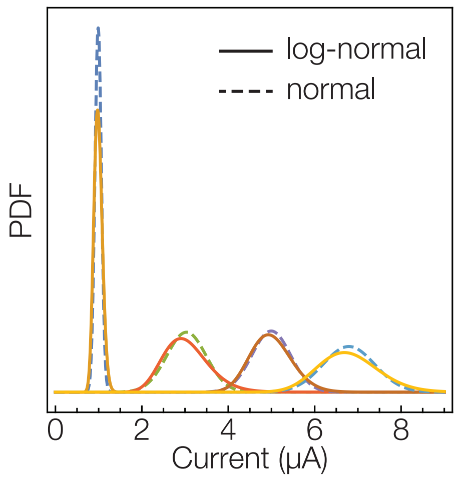

Supplementary Figure 2: **Normal vs. log-normal experimental fit**. Comparison between a normal fit (dashed curves) and a log-normal fit (solid curves) of the four current-based states of the 1T1R hafnium-based devices.

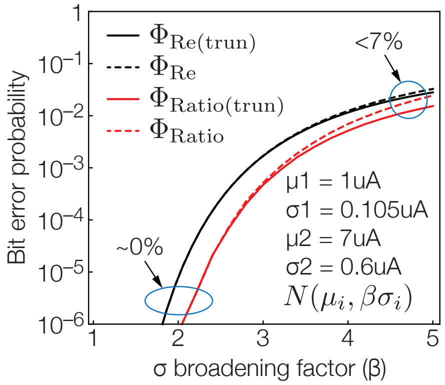

Supplementary Figure 3: **Truncation effect of the BEP of normally distributed states**. Comparison of the BEP of a 2-level memory as a function of the broadening factor β of the states (the effective standard deviation of the normal distributions is $\beta\sigma$). For the lowest and highest resistance states, we assumed the highest and lowest conductance of the experimental data of the 1T1R hafnium-based devices at 100 programming pulses, respectively. The black curves are for the resistance-based encoding. The red curves are for the ratio-based encoding. The solid curves are for truncated distributions, the dashed lines are not truncated. The error in the estimation does not exceed 7%.

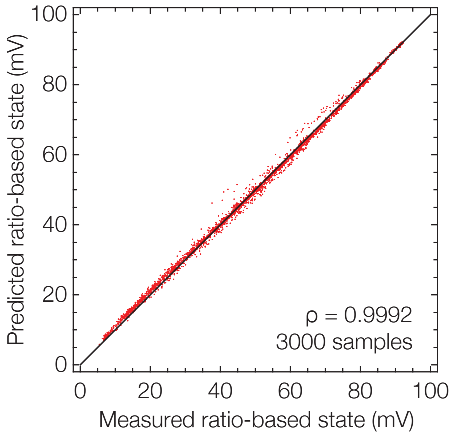

Supplementary Figure 4: **Correlation between the measured and predicted ratio-based states**. The predicted output voltage (computed using the individual resistance of the pair of devices) is compared to the actual voltage measurement of the voltage divider for titanium-oxide devices.
